# A Multi-Step Precision Pathway for Predicting Allograft Survival in Heterogeneous Cohorts of Kidney Transplant Recipients

**DOI:** 10.3389/ti.2023.11338

**Published:** 2023-09-12

**Authors:** Yunwei Zhang, Danny Deng, Samuel Muller, Germaine Wong, Jean Yee Hwa Yang

**Affiliations:** ^1^ Charles Perkins Centre, The University of Sydney, Sydney, NSW, Australia; ^2^ School of Mathematics and Statistics, The University of Sydney, Sydney, NSW, Australia; ^3^ Centre for Kidney Research, Kids Research Institute, The Children’s Hospital at Westmead, Sydney, NSW, Australia; ^4^ Faculty of Medicine and Health, The University of Sydney, Sydney, NSW, Australia; ^5^ School of Mathematical and Physical Sciences, Macquarie University, Sydney, NSW, Australia; ^6^ Sydney School of Public Health, The University of Sydney, Sydney, NSW, Australia; ^7^ Centre for Transplant and Renal Research, Westmead Hospital, Sydney, NSW, Australia; ^8^ Laboratory of Data Discovery for Health Limited (D24H), Hong Kong, Hong Kong SAR, China

**Keywords:** kidney transplantation, kidney transplantation graft survival, allograft survival prediction, prediction model, ANZDATA

## Abstract

Accurate prediction of allograft survival after kidney transplantation allows early identification of at-risk recipients for adverse outcomes and initiation of preventive interventions to optimize post-transplant care. Many prediction algorithms do not model cohort heterogeneity and may lead to inaccurate assessment of longer-term graft outcomes among minority groups. Using data from a national Australian kidney transplant cohort (2008–2017) as the derivation set, we developed P-Cube, a multi-step precision prediction pathway model for predicting overall graft survival in three ethnic subgroups: European Australians, Asian Australians and Aboriginal and Torres Strait Islander Peoples. The concordance index for the European Australians, Asian Australians, and Aboriginal and Torres Strait Islander Peoples subpopulations were 0.99 (0.98–0.99), 0.93 (0.92–0.94) and 0.92 (0.91–0.93), respectively. Similar findings were observed when validating P-cube using an external dataset [Scientific Registry of Transplant Recipient Registry (2006–2020)]. Six sub-categories of recipients with distinct risk factor profiles were identified. Some factors such as blood group compatibility were considered important across the entire transplant population. Other factors such as human leukocyte antigen (HLA)-DR mismatches were unique to older recipients. The P-cube model identifies allograft survival specific risk factors within a heterogenous population and offers personalized survival predictions in a diverse cohort.

## Graphical Abstract

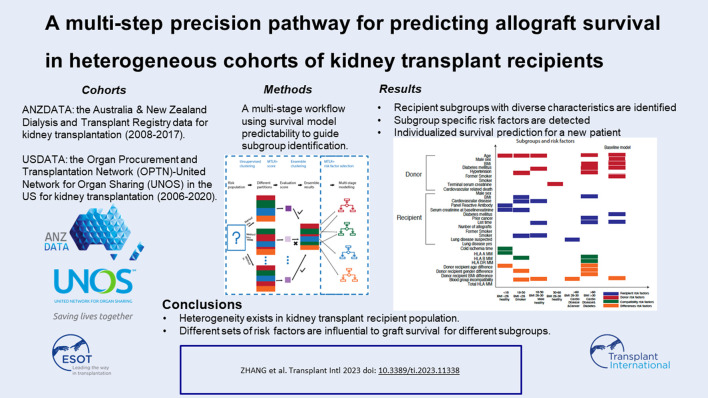

## Introduction

Kidney transplantation confers significant survival and quality of life advantages compared with dialysis for patients with kidney failure [1]. Despite improvements in both short and longer-term allograft survivals in the last two decades, recipients’ survival and quality of life remain inferior compared to the general population, attributed mainly to the complications of immunosuppression including infections, metabolic diseases, and cancer [2]. Maintaining optimal patient and graft survival are therefore the key priorities for transplant recipients, caregivers, and health professionals. Personalized predictions for those at risk of adverse events such as acute rejection, infections, cancer, and allograft loss allow early identification and interventions to optimize clinical care [3]. The derived probabilities of these predictive factors offer unique opportunities for health professionals to target appropriate management options such as immunosuppression strategies at the time of and after transplantation.

Over the past decade, several predictive factors for longer-term graft and patient outcomes have been identified as variables of importance using machine learning-based and traditional regression models [4]. However, prior studies have not accounted for the heterogeneity between subgroups within a transplant cohort [5, 6]. Allograft outcomes are consequences of many pre- and post-transplant events, precipitated by numerous known and unknown factors over the lifespan of a transplant recipient, and may differ between patient characteristics such as age, ethnicity, sex and gender, and other social determinants of health. Knowledge of these factors will guide individualized treatment plans and clinical decision making. Using an established evaluation framework, combined with novel supervised and unsupervised data driven approaches, we first identified the important characteristics that differentiate between distinct recipient subgroups for graft survival predictions. We then developed predictive models for longer-term allograft outcomes within the individual clusters. Finally, we externally validated these models to determine the reliability of their performance characteristics.

## Materials and Methods

### Study Populations

**FIGURE 1:**
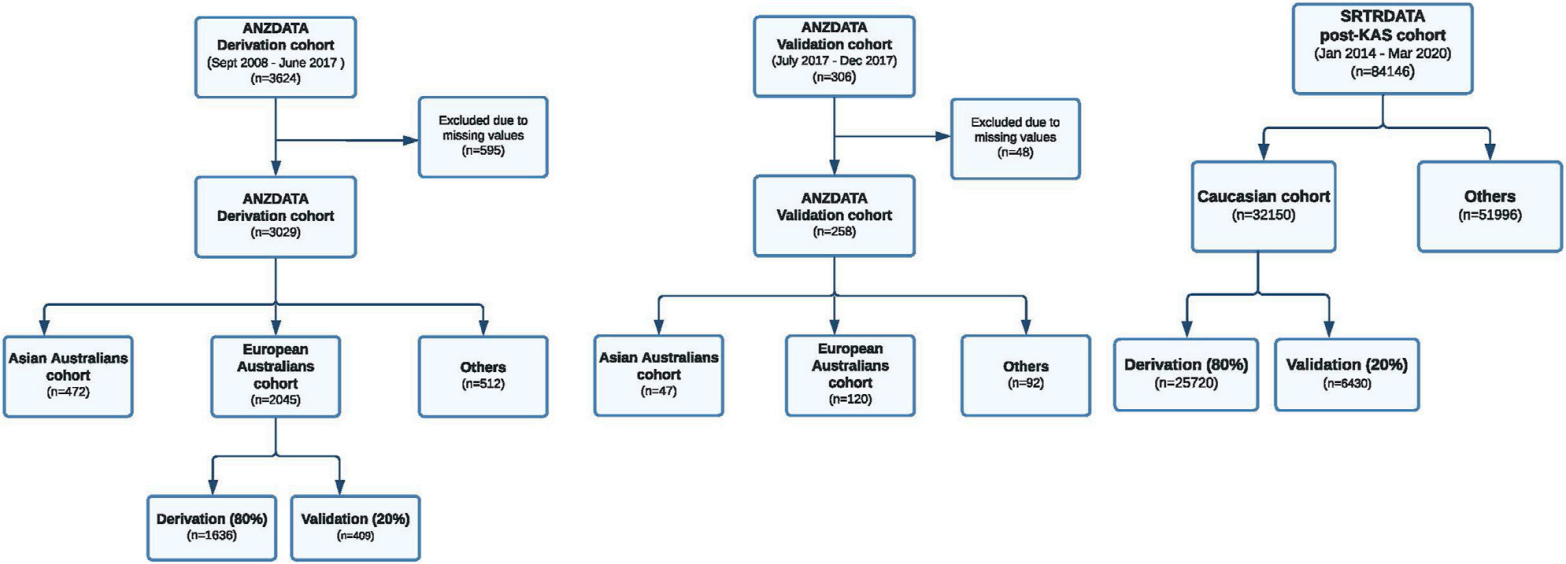
Study flow of the Australian and US derivation and validation cohorts. ANZDATA derivation cohort, ANZDATA validation cohort and the US transplant cohort are shown.

Two separate cohorts, data from all deceased donor kidney transplants within the Australia and New Zealand Dialysis and Transplant Registry (ANZDATA) and the Scientific Registry of Transplant Recipient (SRTR) Registry, were used for the modeling step (Figure 1). The ANZDATA registry includes all kidney transplant recipients between 2008–2017 in Australia and New Zealand. The SRTR registry includes patients transplanted between 2006–2020 in the United States (US). The SRTR database includes data on all donors, waitlisted candidates, and transplant recipients in the US, submitted by the members of the Organ Procurement and Transplantation Network. In this analysis, we selected data from the Australian populations and excluded all donor and recipient data from New Zealand. Data from New Zealand was excluded from the analyses because the deceased donor allocation algorithm (and systems) in New Zealand is different to Australia. There would be significant heterogeneity if both cohorts were combined as a training cohort. We followed 3,624 patients from September 2008 to June 2017 over the median graft survival period of 3.23 years (IQR: 1.79, 5.40 years).

### Study Design

Next, we present a general description of the two different models, a novel multi-step precision pathway and the classical regression model, for the prediction of overall graft survival after kidney transplantation. Epidemiological data have shown that post-transplant outcomes are not uniform for all transplant recipients. Allograft survival differs among gender, ethnic groups, socioeconomic status, and comorbidity status within a heterogenous kidney transplant cohort [7–11]. Therefore, if a group-blind classifier is trained on the entire cohort of transplant recipients for the prediction of allograft outcomes, this classifier will not fit well for all candidates. Rather, the optimal fit will likely apply to the majority, attributed largely to the large sample size, and ignore the minority groups. To address the issue of “fairness” in machine learning [12], we developed a precision prediction pathway (P-cube model) that considers the heterogenous characteristics within different subgroups.

The P-cube model was first developed using data from the European Australian sub cohort. We then assessed the predictive performances of this P-cube model for overall graft survival across all three different ethnic subgroups: European Australians, Asian Australians and Aboriginal and Torres Strait Islander Peoples. To explore the external validity of these models, we tested the modelled algorithm using data from the SRTR registry (*n* = 32,150). Here, we split the data (80:20) into a derivation cohort (*n* = 25,720) and independent validation cohort (*n* = 6,430). The classical regression model was developed using data from the entire Australian derivation cohort and did not account for cohort heterogeneity. We then compared the predictive performances of the P-cube and the classical regression models across all subgroups. The conduct and reporting of this study adhere to the transparent reporting of a multivariable prediction model for individual prognosis or diagnosis (TRIPOD): The TRIPOD statement [13].

### Statistical Analysis

#### Model Building

##### Model I: The Precision Prediction Pathway

The precision prediction pathway (P-cube) model (Supplementary Figure S1) is a hybrid algorithm that incorporates techniques of supervised and unsupervised learnings. The P-cube model consists of two elements. First, we used a “modified consensus unsupervised clustering method” to segregate a heterogeneous population into homogeneous subgroups. Second, for each subgroup, a multi-task logistic regression was applied to determine the risk factors for overall graft survival. Within each subgroup, we estimated the probabilities of graft survival. Specific modeling strategies are detailed in the following.

###### Modified Consensus Unsupervised Clustering

First, we applied a collection of unsupervised clustering approaches (see Supplementary Table S1) such as the K-means and hierarchical clustering methods to define the recipient subgroups. A data-driven ensemble clustering method [14] was used to derive a compilation of stable and robust homogeneous subgroups.

###### Multi-Task Logistic Regression

Using the multi-task logistic regression (MTLR) [15], we determined the risk factors for overall graft survival for transplant recipients within individual subgroups. Implementation of this workflow was performed using R version 4.1.1 and the codes are available at https://github.com/SydneyBioX/P3_model. Variables included in the P-cube model are shown in Supplementary Table S2.

###### Selection of Important Risk Factors for Allograft Survival Within Subgroups

We used the “elbow of the curve” method [16] to determine the important risk factors for overall graft survival. The knee of a curve was defined as a vertex of the graph. This corresponded with the graphical intuition where the curvature has a maximum. Specifically, for each subgroup, the “weights” of the selected risk factors from the MTLR model were ranked from the most to the least important. We then calculated the difference in these weights between two consecutive factors. After visual inspection by a single examiner, a stop line was determined if the differences (i.e., the amount of decrease in the exact weights of the risk factor) were less than a threshold value of 0.007. We have chosen a threshold value of 0.007 because this is the elbow point across all subgroups.

##### Model II: Regression-Based Model

A classical risk modeling strategy was used to build a regression model to determine the risk factors for overall graft survival within the entire derivation cohort, without accounting for recipient and donor heterogeneity.

#### Model Evaluation

We compared the P-cube model predictive performance with the classical regression-based model using Harrell’s C-index [7]. Here, we fitted the classical regression-based and P-Cube models to the independent derivation cohorts and tested the performances of each model using data from the independent internal and external validation cohorts (Figure 1). We examined the stability and the performance of the P-cube model (Model I) using a perturbation strategy, whereby a subset of the derivation cohort was randomly selected (80% of the original cohort) and resampled to create a perturbated P-cube model. The predicted survival probabilities of the original and perturbated P-cube models were compared numerically using Pearson correlation and visually using a scatter plot. We also performed a sensitivity analysis on death censored graft survival using both C-index and the Brier Score. For overall patients’ survival as the outcome of interest, we built the corresponding P-cube model and then assessed its performances.

#### Model Application

To apply the P-cube model in clinical settings, a “model decision tree” was built based on subgroup characteristics. The decision tree allowed us to define the most appropriate prediction pathway and the overall graft survival probability was then estimated for each hypothetical donor-recipient pair.

## Results

### Baseline Characteristics of the Australian and US Cohorts

Within the Australian cohort**,** the average (SD) donor age of the derivation cohort was 48 (16.8) years, with the majority being male (54%), and 26% were from donors of circulatory (DCD). With regards to the recipient characteristics, the mean (SD) age of the derivation cohort was 52 (14.3) years, with the majority being men (65%), and 18% had diabetes at the time of transplantation. Similar characteristics were observed in the independent Australian validation cohort. Within the US cohort, the average (SD) donor age of the derivation cohort was 38 (15.4) years, with most of these deceased donors being male (62%). The mean recipient (SD) age of the derivation cohort was 54 (15.5) years, with the majority being men (61%), and 31% of recipients had diabetes mellitus at the time of transplantation. Similar characteristics were observed in the independent US validation cohort (Supplementary Tables S3, S4)**.**

### Prediction Performances of the Classical Regression and P-Cube Models

For the classical regression model, the concordance index (C-index) (95% CI) was highest if the model was applied to the European Australian cohort: 0.95 (0.93–0.96), followed by the Asian Australian cohort: 0.87 (0.86–0.88) and the Aboriginal and Torres Strait Islander Peoples cohort: 0.78 (0.76–0.80). For the P-cube model, the C-index for the European Australians, Asian Australians, and Aboriginal and Torres Strait Islander Peoples cohorts were 0.99 (0.98–0.99), 0.93 (0.92–0.94) and 0.92 (0.91–0.93), respectively. The P-cube model was robust to small perturbations (Supplementary Figure S2). The Pearson correlation between the predicted survival probabilities using the original P-cube and the perturbed P-cube model was 0.92. The Brier Score (a lower score indicates better performance) for 5 years post-transplant using the classical regression model for the three cohorts compared with the P-cube model are as following: 0.217 vs. 0.216 (European Australians), 0.116 vs. 0.115 (Asian Australians), 0.218 vs. 0.216 (First Nation Peoples). Similarly, for 10 years post-transplant, the Brier Scores are 0.336 vs. 0.330 (European Australians), 0.124 vs. 0.123 (Asian Australians), 0.354 vs. 0.348 (First Nation Peoples). In our sensitivity analysis, we also found P-cube outperformed the classical regression model evaluated by both the C-index and Brier Score (Supplementary Tables S6, S7) for death censored graft and overall patient survivals. Similar recipient subgroups and risk factors were identified for patients’ overall survival, indicating that patients’ overall health level is critical for both allograft survival and post-transplant recovery (details can be found in Supplementary Tables S6, S7).

### Defining the Individual Subgroups Using the P-Cube Model

**FIGURE 2:**
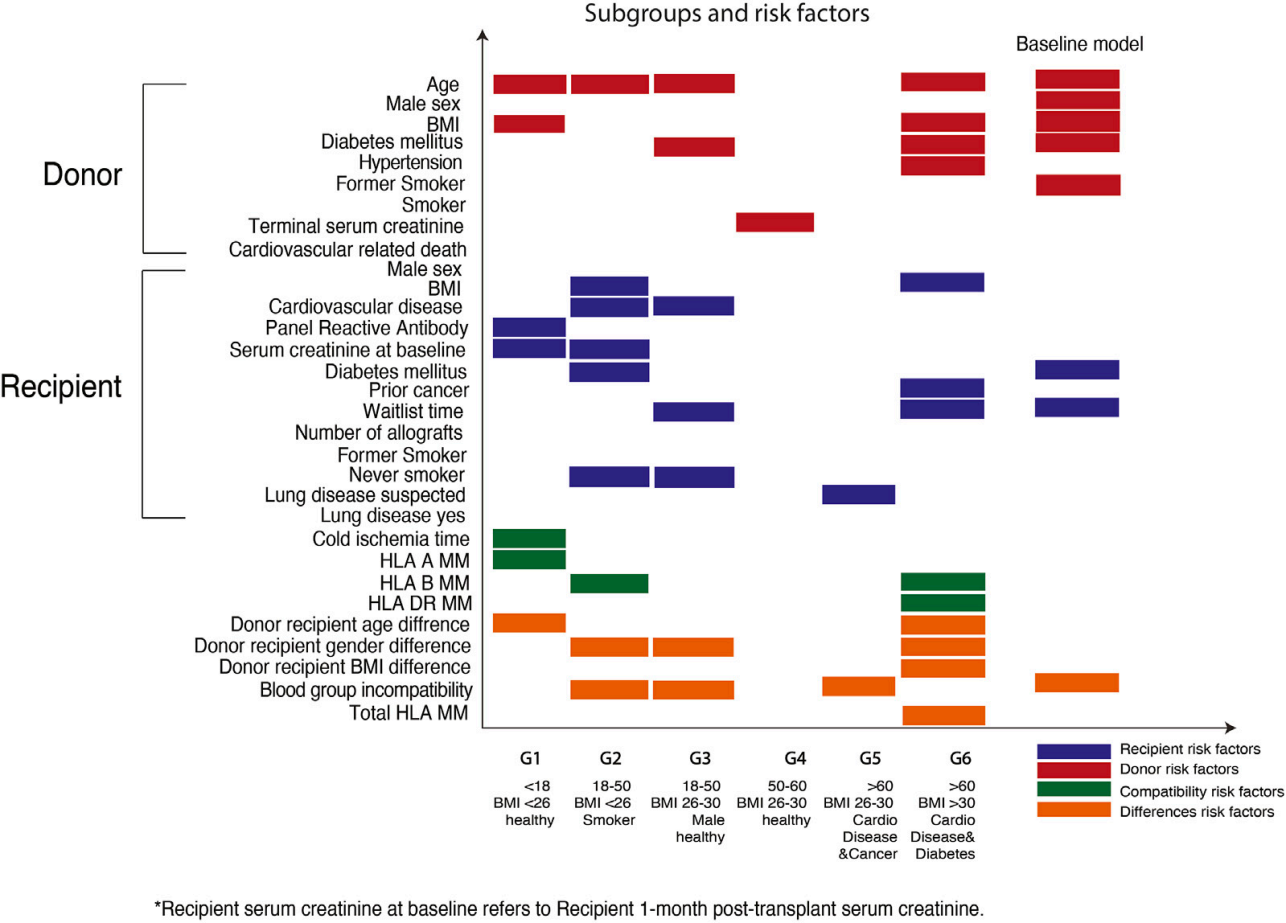
Defining subgroups and the predictive factors for graft survivals within the Australian heterogenous populations. *X*-axis shows the six subgroups defined within the Australian transplant populations. *Y*-axis shows the predictive factors corresponding to each subgroup.

Using an unsupervised data driven approach, six subgroups with unique recipient characteristics were identified (Figure 2). Each subgroup had unique features, including recipient age, comorbidities, and demographics. For example, group 1 included predominantly young transplant recipients (less than 18 years) and group 6 comprised of older recipients with comorbidities such as cardiovascular disease and diabetes mellitus.

### Risk Factors for Allograft Survival Within Subgroups

**FIGURE 3:**
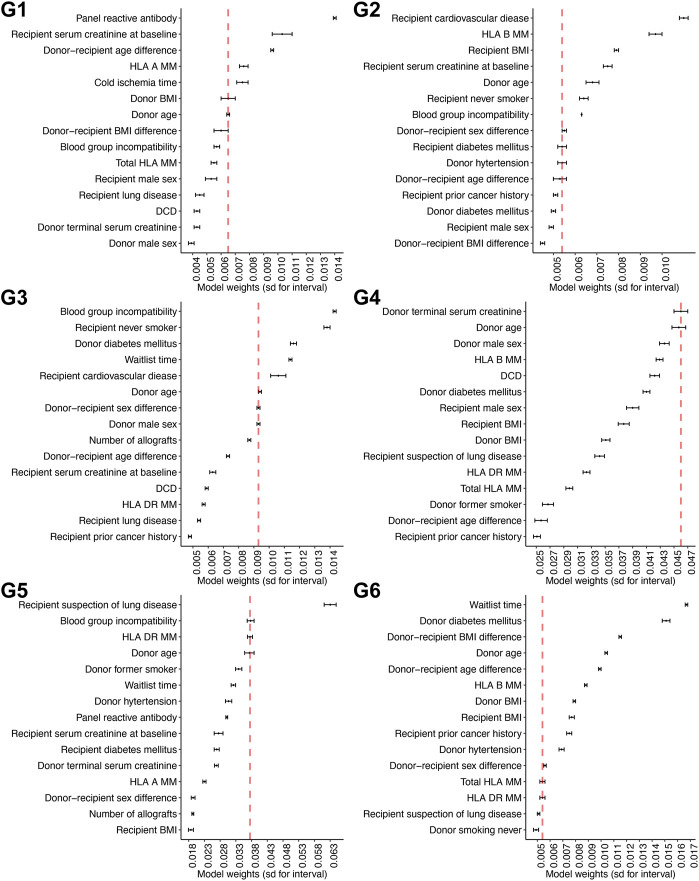
Defining the weights for the individual predictive factors across the different subgroups Weights for each particular predictive factor within each subgroup are shown in this figure. Six panels are corresponding to six models for six subgroups.

Using the elbow of the knee method (Figure 3), we identified the common risk factors for overall graft survival across all subgroups, and these included donor age and donor-recipient blood group compatibility. Moreover, unique predictive factors were also observed within the heterogenous subgroups. Within the pediatric sub cohort, donor age, recipient sensitization status (defined as panel reactive antibody), and donor-recipient age differences were the most important factors for allograft survival. Among the older recipients and those with comorbidities, human leukocyte antigen (HLA) DR mismatches were most predictive for overall graft survival.

### External Validation Using Data From the SRTR Registry

When applied the modelling to the US cohort, the C-indices for the P-cube and classical regression models were 0.84, and 0.83, respectively. Within specific ethnic subgroups in the US, the predictive performances were comparable across the White and Asian sub-populations. Similarly, we also identified 8 subgroups within the US cohort (Supplementary Figure S3). Of all known risk factors, recipient-donor age difference was the most important predictive factor for overall graft survival within a sub-cohort (middle-aged recipient with comorbidities).

### Applying the P-Cube Model in Clinical Settings

To test the application of the P-cube model in “real-life” settings, we applied the algorithm in three hypothetical transplant candidates with different characteristics (Supplementary Figures S4, S5). Three distinct predictive pathways were identified. The red curve represents a female paediatric recipient (aged 16 years) from subgroup 1 (pathway M1 in Supplementary Figure S4). The green curve represents a 45 years old male recipient without any major comorbidities such as cardiovascular or lung disease from subgroup 3, pathway M3 in Supplementary Figure S4. Lastly, the blue curve represents a 62 years old female recipient with diabetes and cardiovascular disease at the time of transplantation from subgroup 6 (pathway M6 in Supplementary Figure S4). The key factors that determined allograft survival in a young candidate were donor-recipient age differences, donor age and sensitization status of the recipient. In contrast, immunological mismatches and other recipients’ characteristics such as co-existing cardiovascular disease and diabetes mellitus were predictive of overall graft survival in older candidates with comorbidities.

## Discussion

Most of the published approaches to predict allograft and patient survival after transplantation use a one-size fits-all-model to apply to the entire transplant population and do not capture the heterogeneity within the population of interests. Many of these models construct a single risk score and apply it to the whole population without considering the nuances and the risk profiles of the individuals. Using data from the Australian kidney transplant population, we developed a novel prediction pathway using combined supervised and unsupervised data driven approaches to allow personalized prediction for allograft survivals in a heterogeneous cohort of kidney transplant recipients. The P-cube model has good discriminative power across all subgroups in the Australians cohort with improved predictive ability, particularly for minority groups such as our Aboriginal and Torres Strait Islander Peoples, when compared with the classical regression model. We have also demonstrated robustness and external validity of our modelling with good predictive ability for allograft survivals within the US transplant populations. Another novel aspect of the P-cube model is its ability to segregate and characterize the predictive factors within a homogenous subgroup. Our model recognized some of the features such as donor age that are consistently and equally important across all subgroups, while some factors such as HLA-DR mismatches and sensitization status are unique to certain membership within individual subgroups. Thus, allowing accurate survival predictions for patients and families in real time.

The P-cube model provides an opportunity for personalised prediction of longer-term allograft survival in kidney transplant recipients. Prior models depend largely on static and one-dimensional data at fixed time points and fixed covariates. The P-cube model is a flexible platform that allows identification of individuals who may be at a higher risk of experiencing allograft loss. This in turn allows clinicians to provide a more accurate prognosis for patients as well as potential for early intervention (such as modification of immunosuppression or to instigate other monitoring strategies). Understanding patients’ graft and patient prognoses will facilitate access to certain services and benefits. In addition, knowledge of the transplant recipients’ predicted long-term outcomes provides an opportunity to refine our allocation algorithm, with consideration of both donor and recipient characteristics to facilitate appropriate allocation pathways to maximise efficiency and efficacy of transplantation. Finally, it is important to emphasise that this model is not only limited to kidney transplant recipients, but can also be applied to other solid organ transplant recipients with input of appropriate variables.

Our P-cube model can be applied to the assessment of other subcategories across different transplant settings. Using an array of unsupervised learning approaches (partitioned and hierarchical-base methods), the P-cube model allows integration of other non-traditional clinical risk factors such as molecular immunological data (such as eplet mismatches or T cell epitope predictions) to allow for personalized risk predictions. The P-cube model can also handle regression, classification, and survival analysis in a streamlined algorithm. Our model can also be easily re-trained as new information becomes available and when clinical practices change with time.

Our modeling approaches, however, have several potential limitations. The computational time for this combined supervised and unsupervised learning strategy is lengthy and may take up to 24 h for processing time with currently available standard desktop computing. In future work, the selection of the threshold values for the determination of important risk factors for graft survival could be examined further as well as other methods such as bootstrapping and permutation tests. The ANZDATA registry does not routinely collect anti-HLA donor specific antibodies. Having access to these additional immunological data may enhance the model performance. We have validated the model in a single external validation dataset and assessed its performance within ethnics subgroups. Future research should test this algorithm in other subpopulations including different genders and socioeconomic groups.

In conclusion, we have developed a multistep prediction tool for allograft survival to guide clinical-decision making within a heterogenous cohort of kidney transplant recipient. This model can be extended to include other time to event endpoints such as patient and cause-specific survivals and acute rejection in future iterations. Findings derived from the P-cube model will provide health professionals and patients the relevant prognostic information to guide treatment decisions and contribute to personalized care.

## Data Availability

Publicly available datasets were analyzed in this study. This data can be found here: https://www.anzdata.org.au/anzdata/.

## Abbreviations

ANZDATA, Australia and New Zealand Dialysis and Transplant Registry; C-index, concordance index; HLA, human leukocyte antigen; MTLR, multi-task logistic regression; P-Cube, multi-step precision prediction pathway; SRTR, Scientific Registry of Transplant Recipient.
