# Supplementary material for: A Multi-Step Precision Pathway for Predicting Allograft Survival in Heterogeneous Cohorts of Kidney Transplant Recipients
Source: Transpl Int. 2023 Sep 12;36:11338. doi: 10.3389/ti.2023.11338 (PMC10520244; doi:10.3389/ti.2023.11338)
Supplement: Supplementary file 1 [file DataSheet1.docx]

**Supplementary Table 1. Clustering methods used in the P-cube model where the number of groups varied from 2 to 30**

| **Category** | **Clustering method name** | **Input data format** | **Distance calculation methods** |
| --- | --- | --- | --- |
| Model based | Latent class analysis | Original | NA |
| Partition based | Partition around medoids | Original | Gower |
|  |  | Numerical | Euclidean |
|  |  | Scaled numerical | Euclidean |
| Hierarchy based | Hierarchical clustering with Wald linkage | Original | Gower |
|  |  | Numerical | Euclidean |
|  |  | Scaled numerical | Euclidean |
|  | Hierarchical clustering with average linkage | Original | Gower |
|  |  | Numerical | Euclidean |
|  |  | Scaled numerical | Euclidean |

**Supplementary Table 2. Variables included in the P-cube model**

| **Step** | **Data** | **Variable names in the data** |
| --- | --- | --- |
| **One** | Australian Cohort | Recipient: Male sex, cardiovascular disease, blood group, diabetes mellitus, prior cancer, age, BMI, smoking status, lung disease |
|  | US Cohort | Recipient: Male sex, cardiovascular disease, blood group, diabetes mellitus, prior cancer, age, BMI, hepatitis status |
| **Two** | Australian Cohort | Donor: Age, male sex, BMI, diabetes mellitus, hypertension, smoking status, terminal serum creatinine, DCD  Recipient: Male sex, BMI, cardiovascular disease, Panel Reactive Antibody, serum creatinine at baseline, diabetes mellitus, prior cancer, waitlist time, number of allografts, cold ischemia time, HLA A mismatch, HLA B mismatch, HLA DR mismatch, smoking status, lung disease  Donor recipient age differences, donor recipient sex differences, donor recipient BMI differences, blood group incompatibility |
|  | US Cohort | Donor: Age, male sex, BMI, diabetes mellitus, hypertension, smoking status, terminal serum creatinine, DCD  Recipient: Male sex, BMI, cardiovascular disease, Panel Reactive Antibody, serum creatinine at baseline, diabetes mellitus, prior cancer, waitlist time, number of allografts, cold ischemia time, HLA A mismatch, HLA B mismatch, HLA DR mismatch, hepatitis status  Donor recipient age differences, donor recipient sex differences, donor recipient BMI differences, blood group incompatibility |

**Supplementary Table 3.**

**Baseline characteristics of the Australian cohorts**

|  | **Validation**  **n = 409** | **Train**  **n = 1,636** | **External Validation**  **n = 120** |
| --- | --- | --- | --- |
| **Donor characteristics** |  |  |  |
| **Age,** mean (SD) |  | 47.9 (16.8) | 49.0 (14.7) |
| **Male sex,** n (%) | 207 (51) | 905 (55) | 63 (52) |
| **BMI,** kg/m^2^ (SD) | 27.2 (6.3) | 27.3 (6.1) | 27.5 (6.0) |
| **Diabetes Mellitus,** n (%) | 27 (6.6) | 109 (6.7) | 13 (11) |
| **Hypertension,** n (%) | 110 (27) | 455 (28) | 28 (23) |
| **Smoking Status,** n (%) |  |  |  |
| Former | 88 (22) | 393 (24) | 22 (18) |
| Never | 151 (37) | 601 (37) | 42 (35) |
| Unknown | 1 (0.2) | 2 (0.1) | 0 (0) |
| **Terminal serum creatinine,** mean (SD) | 88.4 (66.3) | 89.73 (67.0) | 92.47 (92.0) |
| **DBD,** n (%) | 314 (77) | 1,203 (74) | 79 (66) |
| **DCD,** n (%) | 95 (23) | 433 (26) | 41 (34) |

**DCD – death from circulatory death, #DBD – death from brain death*

|  | **Validation**  **n = 409** | **Train**  **n = 1,636** | **External Validation**  **n = 120** |
| --- | --- | --- | --- |
| **Recipient Characteristics** |  |  |  |
| **Age,** mean (SD) | 51.7 (14.3) | 51.9 (14.3) | 52.6 (13.7) |
| **Male sex,** n (%) | 267 (65) | 1,061 (65) | 82 (68) |
| **BMI,** kg/m^2^ (SD) | 27.4 (5.7) | 27.6 (5.7) | 28.1 (6.4) |
| **Cardiovascular Disease,** n (%) | 85 (21) | 342 (21) | 25 (21) |
| **Panel Reactive Antibody,** mean (SD) | 6.6 (17.5) | 8.5 (20.6) | 18.8 (33.4) |
| **Serum Creatinine at baseline,** mean (SD) | 708.3 (288.6) | 714.7 (301.3) | 693.7 (291.9) |
| **Diabetes Mellitus,** n (%) | 54 (13) | 288 (18) | 23 (19) |
| **Prior Cancer,** n (%) | 28 (6.8) | 110 (6.7) | 19 (16) |
| **Waitlist time,** days (SD) | 564.1 (628.9) | 536.02 (578.8) | 656.9 (820.1) |
| **Number of allografts,** n (%) |  |  |  |
| 1 | 400 (98) | 1,591 (97) | 109 (91) |
| 2 | 9 (2.2) | 44 (2.7) | 11 (9.2) |
| **Smoking status,** n (%) |  |  |  |
| Former | 160 (39) | 599 (37) | 51 (42) |
| Never | 201 (49) | 861 (53) | 52 (43) |
| Unknown | 1 (0.2) | 0 (0) | 0 (0) |
| **Lung disease,** n (%) | 17 (4.2) | 115 (7.0) | 13 (11) |
| **Cold ischemia time,** hours (SD) | 11.0 (4.3) | 11.3 (4.5) | 11.2 (4.7) |
| **#HLA A MM,** n (%) |  |  |  |
| 0 | 83 (20) | 318 (19) | 19 (16) |
| 1 | 203 (50) | 766 (47) | 61 (51) |
| 2 | 123 (30) | 552 (34) | 40 (33) |
| **HLA B MM,** n (%) |  |  |  |
| 0 | 77 (19) | 272 (17) | 24 (20) |
| 1 | 163 (40) | 676 (41) | 45 (38) |
| 2 | 169 (41) | 688 (42) | 51 (42) |
| **HLA DR MM,** n (%) |  |  |  |
| 0 | 141 (34) | 537 (33) | 38 (32) |
| 1 | 129 (32) | 580 (35) | 40 (33) |
| 2 | 139 (34) | 519 (32) | 42 (35) |

*#HLA – human leukocyte antigen mismatches*

**Supplementary Table 4.**

**Baseline characteristics of the US cohorts**

|  | **Derivation**  **n = 25,7201** | **Validation**  **n = 6,4301** |
| --- | --- | --- |
| **Donor characteristics** |  |  |
| **Age,** mean (SD) | 38.1 (15.4) | 38.2 (15.2) |
| **Male sex,** n (%) | 15,826 (62) | 3,986 (62) |
| **BMI,** kg/m^2^ (SD) | 28.0 (7.1) | 28.0 (7.1) |
| **Diabetes Mellitus,** n (%) | 1,816 (7.1) | 453 (7.0) |
| **Hypertension,** n (%) | 6,871 (27) | 1,698 (26) |
| **Smoking Status,** n (%) |  |  |
| Never | 19,998 (78) | 4,987 (78) |
| Unknown | 376 (1.5) | 94 (1.5) |
| Current | 5,346 (21) | 1,349 (21) |
| **Terminal serum creatinine,** mean (SD) | 1.21 (1.1) | 1.21 (1.1) |
| **DCD,** n (%) | 5,694 (22) | 1,417 (22) |
| **DBD,** n (%) | 20,026 (78) | 5013 (78) |

**DCD – death from circulatory death, #DBD – death from brain death*

|  | **Derivation**  **n = 25,7201** | **Validation**  **n = 6,4301** |
| --- | --- | --- |
| **Recipient Characteristics** |  |  |
| **Age,** mean (SD) | 53.7 (15.5) | 53.9 (15.4) |
| **Male sex,** n (%) | 15,640 (61) | 3,886 (60) |
| **BMI,** kg/m^2^ (SD) | 28.1 (5.7) | 28.2 (5.7) |
| **Cardiovascular Disease,** n (%) | 2,425 (9.4) | 589 (9.2) |
| **Panel Reactive Antibody,** mean (SD) | 23.81 (36.9) | 23.80 (36.9) |
| **Serum Creatinine at baseline,** mean (SD) | 7.12 (3.4) | 7.11 (3.5) |
| **Diabetes Mellitus,** n (%) | 7,940 (31) | 2,021 (31) |
| **Prior Cancer,** n (%) | 3,571 (14) | 864 (13) |
| **Waitlist time,** days (SD) | 773.5 (789.7) | 754.0 (784.4) |
| **Number of allografts,** n (%) |  |  |
| 0 | 22,014 (86) | 5,466 (85) |
| 1 | 3,182 (12) | 843 (13) |
| 2 | 466 (1.8) | 108 (1.7) |
| 3 | 55 (0.2) | 11 (0.2) |
| 4 | 3 (<0.1) | 2 (<0.1) |
| **Cold ischemia time,** hours (SD) | 17.00 (8.6) | 16.97 (8.7) |
| **HLA A MM,** n (%) |  |  |
| 0 | 4,299 (17) | 1,085 (17) |
| 1 | 10,540 (41) | 2,629 (41) |
| 2 | 10,881 (42) | 2,716 (42) |
| **HLA B MM,** n (%) |  |  |
| 0 | 3,050 (12) | 786 (12) |
| 1 | 7,283 (28) | 1,842 (29) |
| 2 | 15,387 (60) | 3,802 (59) |
| **HLA DR MM,** n (%) |  |  |
| 0 | 5,648 (22) | 1,442 (22) |
| 1 | 12,023 (47) | 2,982 (46) |
| 2 | 8,049 (31) | 2,006 (31) |

*#HLA – human leukocyte antigen mismatches*

**Supplementary Table 5. US cohort P-cube performances on different ethnicity groups.**

| **Model** | **Single risk model** | **P-cube model** |
| --- | --- | --- |
| White | 0.839 (0.838,0.839) | 0.844 (0.843,0.844) |
| Asian | 0.844 (0.843,0.844) | 0.841 (0.841,0.841) |
|  |  |  |

**Results:**

Here, we show the P-cube performance for the US cohort on different ethnicity groups. P-cube is able to achieve a high C-index for the White cohort but is only able to achieve a comparable C-index for the Asian sub-populations.

**Supplementary Table 6. Australian cohort P-cube model with death-censored allograft survival as outcomes.**

| Model | C-index | 5-yr Brier Score | 10-yr Brier Score |
| --- | --- | --- | --- |
| P-cube | 0.864(0.863,0.865) | 0.198 | 0.183 |
| Classical model | 0.809(0.807,0.810) | 0.193 | 0.193 |

**Supplementary Table 7. Australian cohort P-cube model with patients’ overall survival as outcomes.**

| Model | C-index | 5-yr Brier Score | 10-yr Brier Score |
| --- | --- | --- | --- |
| P-cube | 0.854(0.849,0.858) | 0.136 | 0.360 |
| Classical model | 0.831(0.825,0.837) | 0.136 | 0.363 |

**Supplementary Figure 1. P-cube model.**


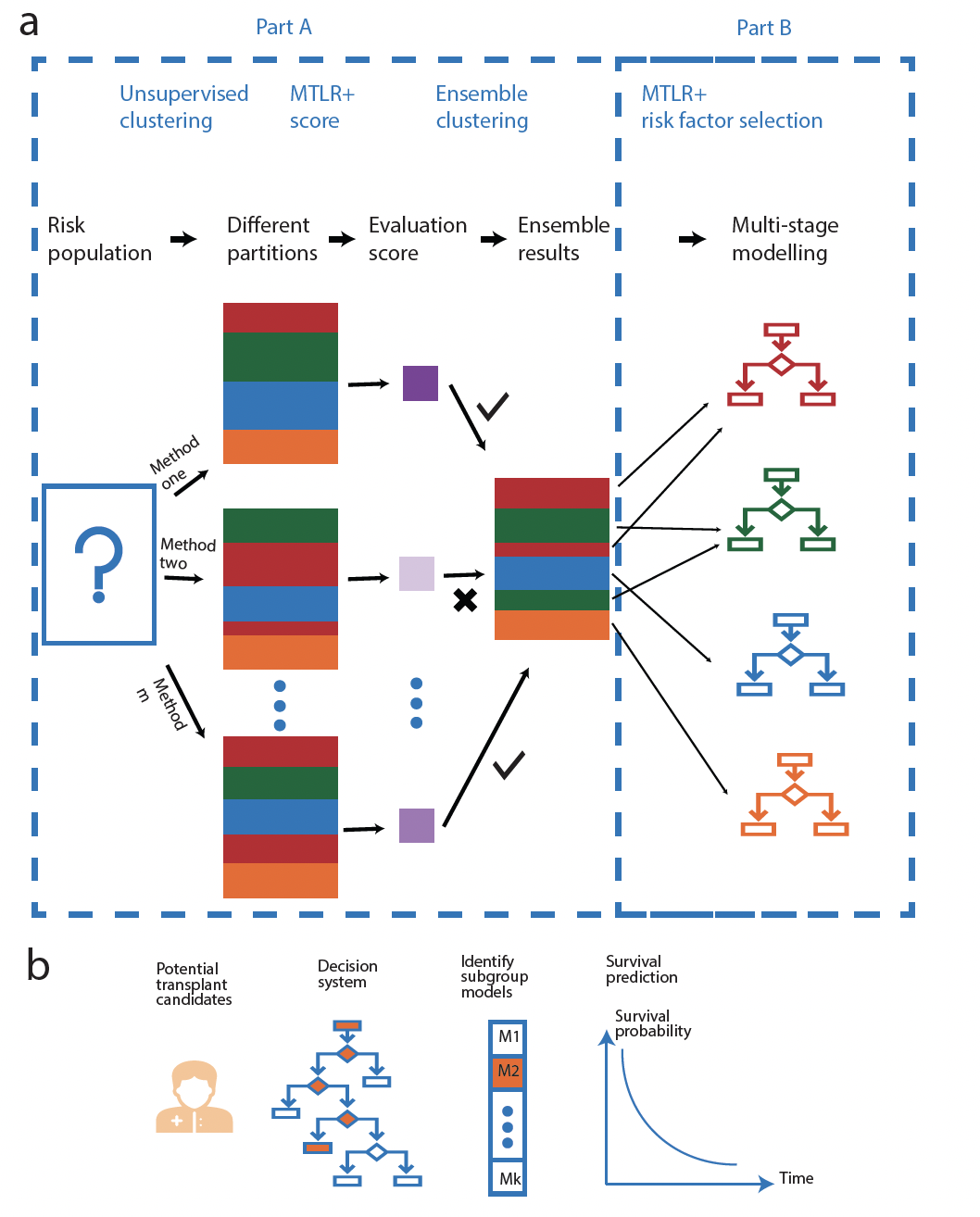


**Supplementary Figure 2. Comparison of the predicted survival probabilities using the P-cube and the perturbated P-cube models.**

**
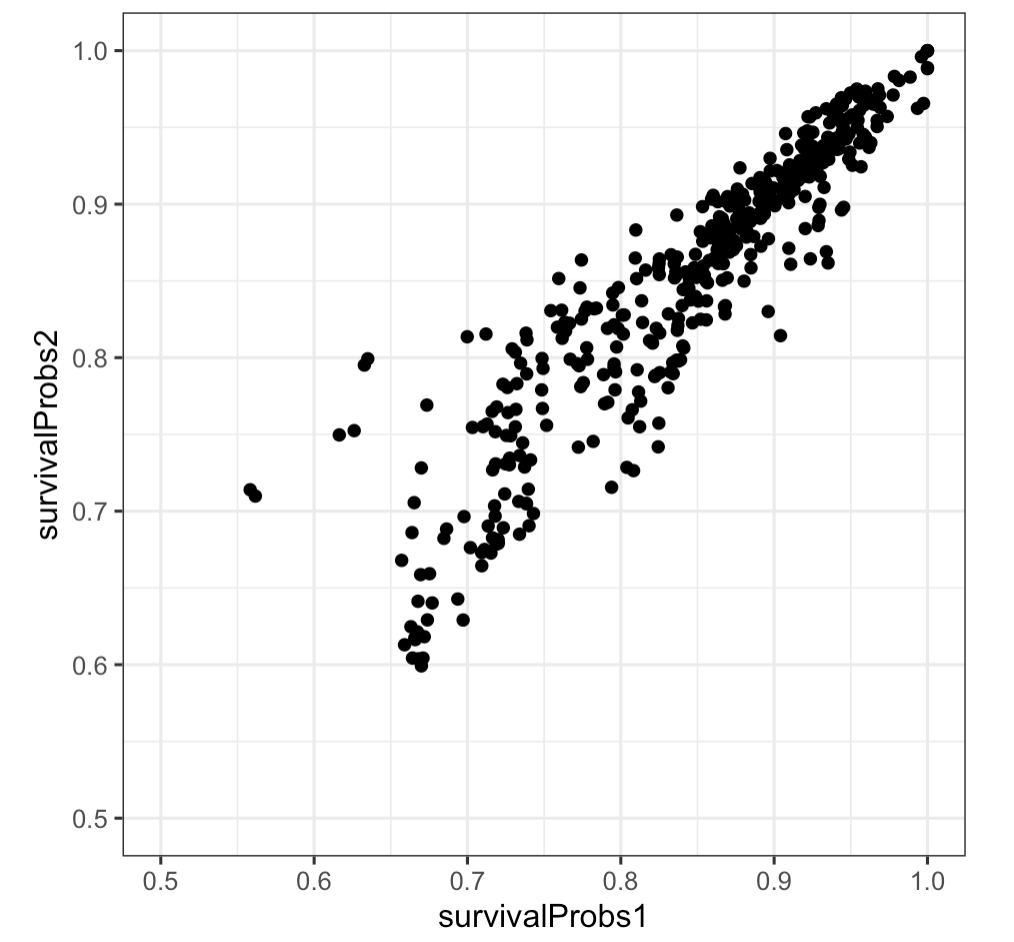
**

**Supplementary Figure 3. Defining subgroups and the predictive factors for graft survivals within the US heterogenous populations**


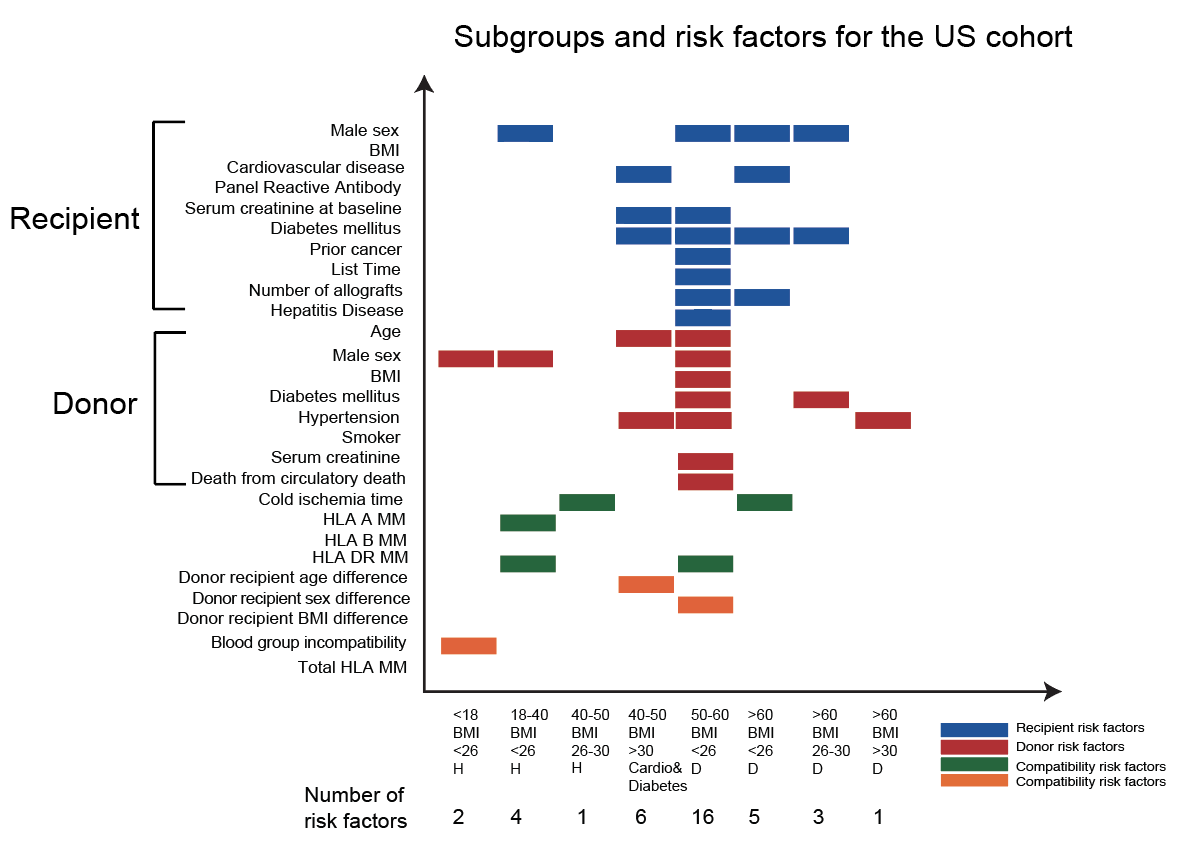


**Supplementary Figure 4. Prediction pathway for the Australian cohort**


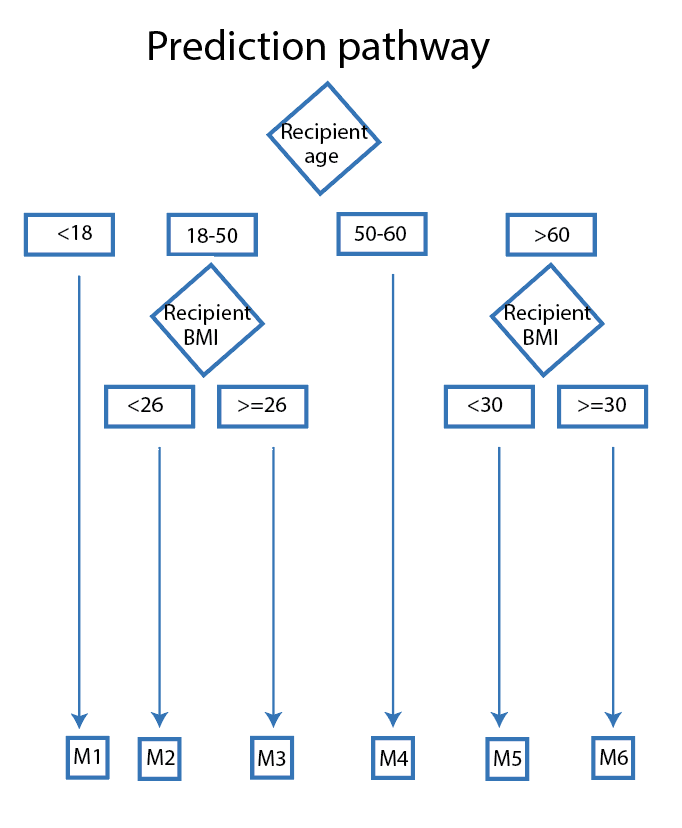


**Supplementary Figure 5. Model application: applying the prediction pathway for individual transplant candidates**

**
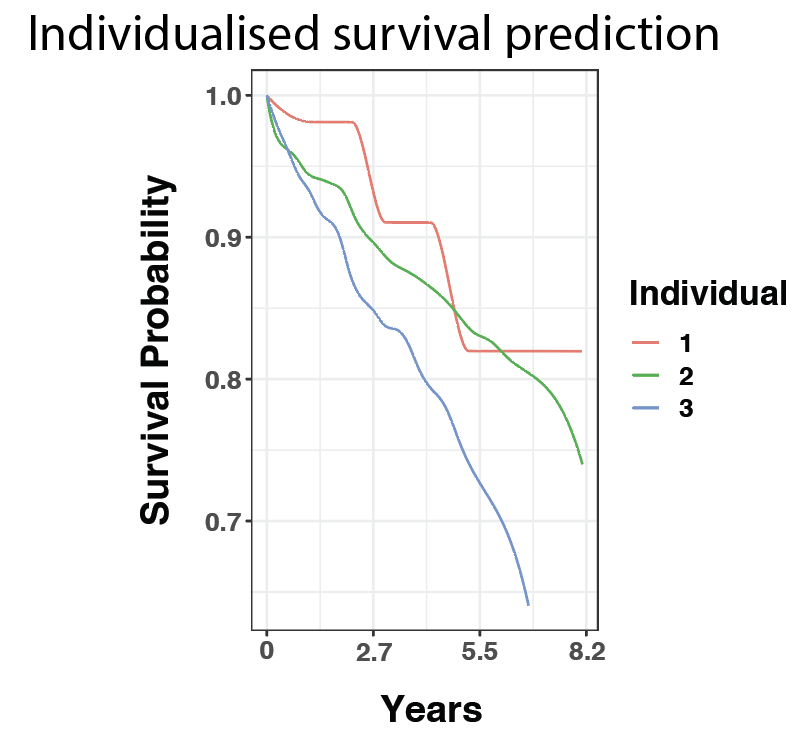
**

**Supplementary Figure 6. Defining subgroups and subgroup specific factors for patients’ overall survival.**


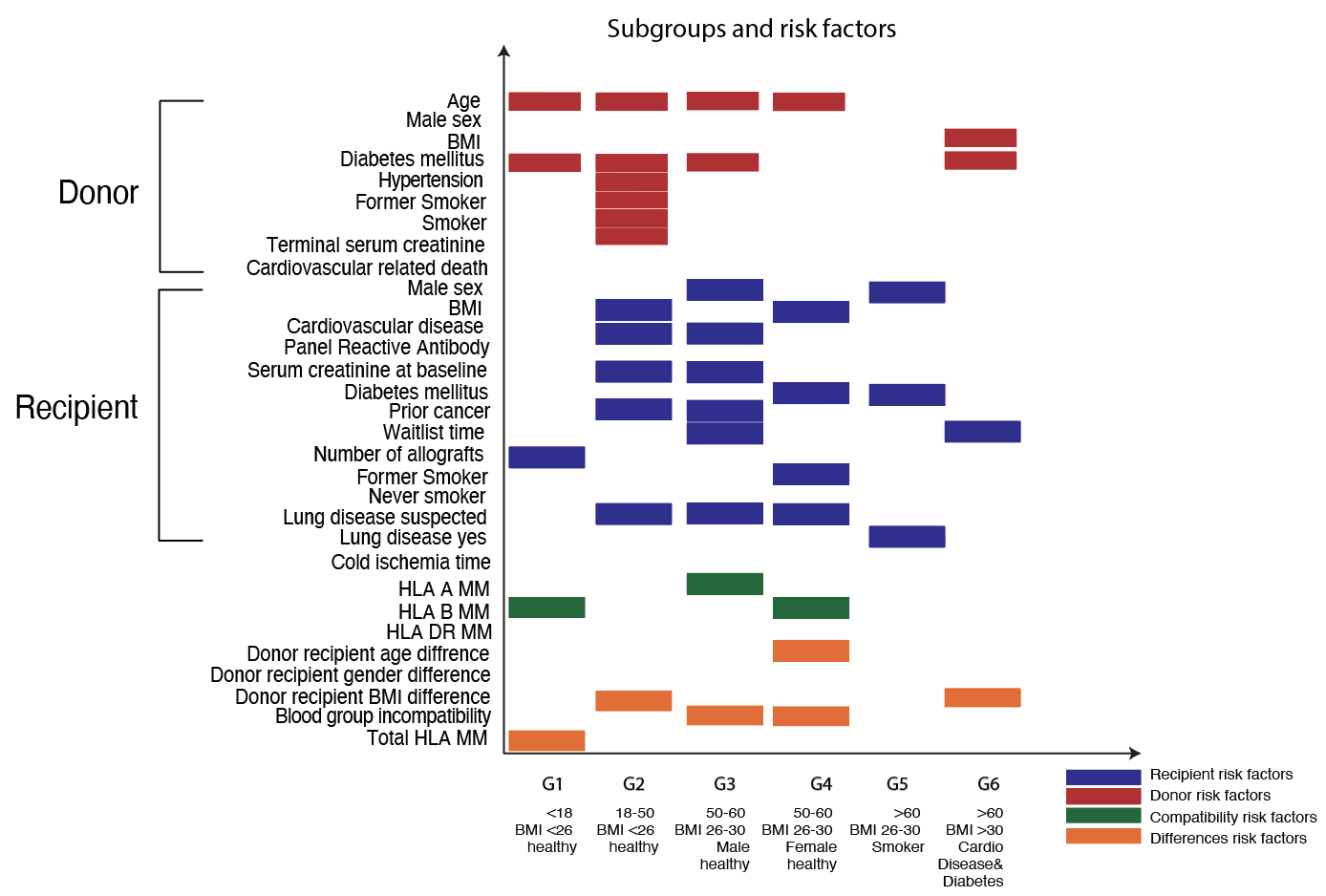


**Supplementary Figure 7. Weights for subgroup specific risk factors for patients’ overall survival.**


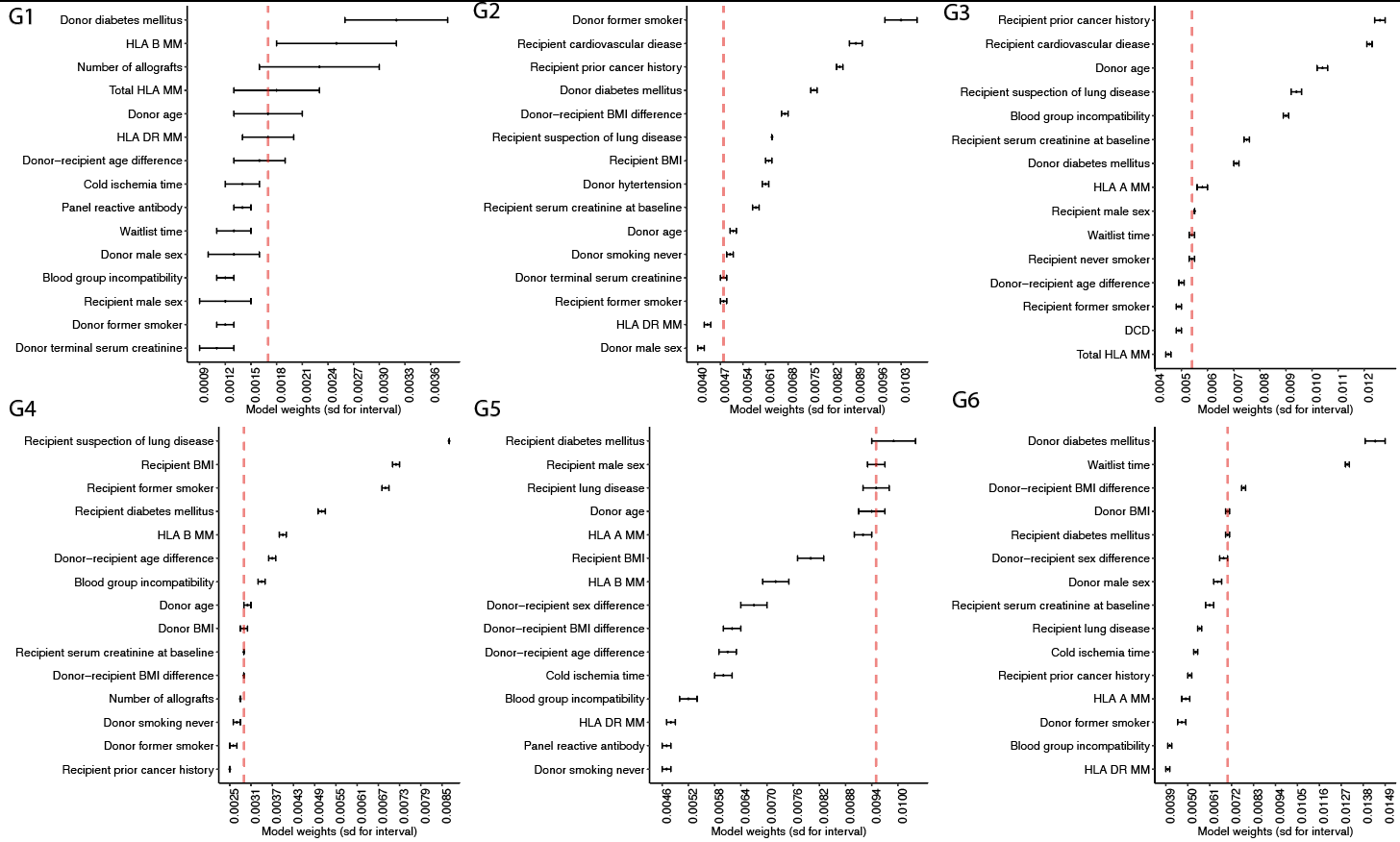


**Results:**

We obtained 6 subgroups with slightly different characteristics compared to using graft survival as the outcome. One 18-50 healthy subgroup was identified, and two 50-60 healthy groups were identified with one is a female group and the other is a male group. For recipients aged greater than 60, similar with before, we have one group with cardio disease and diabetes mellitus and another group is a non-diabetes group. The associated subgroup specific risk factors are shown in Supplementary Figure 6. Similarly, risk factors such as donor age are important across the entire transplant population. Other risk factors such as number of allografts is unique to one subgroup (paediatric group here). Our Supplementary Figure 7 illustrates exact weights for those subgroup specific risk factors by each subgroup.
